# Caracterização Fisiológica de Endotipos Coronários: Possíveis Ligações com Isquemia Miocárdica

**DOI:** 10.36660/abc.20250340

**Published:** 2026-01-09

**Authors:** Lucía Matute-Blanco, Thalia Belmonte, Kristian Rivera, Marcos García-Guimaraes, Juan Casanova-Sandoval, Iván D. Benítez, Georgina Fuertes-Ferre, Ainhoa Pérez Guerrero, Raúl Millán Segovia, Fernando Worner, David De Gonzalo-Calvo, Diego Fernández-Rodríguez

**Affiliations:** 1 Arnau de Vilanova University Hospital Lleida Espanha Arnau de Vilanova University Hospital, Lleida – Espanha; 2 IRBLleida Grup de Fisiologia i Patologia Cardiaca Lleida Espanha IRBLleida - Grup de Fisiologia i Patologia Cardiaca, Institut de Recerca Biomèdica de Lleida Fundació Dr. Pifarré, Lleida – Espanha; 3 IRBLleida Translational Research in Respiratory Medicine Group Lleida Espanha IRBLleida - Translational Research in Respiratory Medicine Group, Institut de Recerca Biomèdica de Lleida Fundació Dr. Pifarré, Lleida – Espanha; 4 CIBERES Instituto de Salud Carlos III Madrid Espanha Centro de Investigación Biomédica en Red de Enfermedades Respiratorias (CIBERES), Instituto de Salud Carlos III, Madrid – Espanha; 5 Hospital Universitario de Cabueñes Gijón Asturias Espanha Hospital Universitario de Cabueñes, Gijón, Asturias – Espanha; 6 Lleida Biomedical Research Institute University of Lleida Lleida Espanha Department of Basic Medical Sciences, Lleida Biomedical Research Institute (IRBLleida), University of Lleida, Lleida – Espanha; 7 Hospital Universitario Miguel Servet Department of Cardiology Zaragoza Espanha Hospital Universitario Miguel Servet - Department of Cardiology, Zaragoza – Espanha; 8 Hospital Clínico Universitario Lozano Blesa Department of Cardiology Zaragoza Espanha Hospital Clínico Universitario Lozano Blesa - Department of Cardiology, Zaragoza – Espanha; 9 Hospital Universitario Son Espases Department of Cardiology Palma Espanha Hospital Universitario Son Espases - Department of Cardiology, Palma – Espanha

**Keywords:** Circulação Coronária, Doença da Artéria Coronariana, Reserva Fracionada de Fluxo Miocárdico

## Abstract

**Fundamento:**

A caracterização fisiológica abrangente dos endotipos coronários permanece limitada, especialmente na disfunção epicárdica.

**Objetivos:**

Descrever os perfis hemodinâmicos em todo o espectro de endotipos coronários e explorar possíveis ligações com a isquemia.

**Métodos:**

Pacientes com suspeita de síndromes coronárias crônicas submetidos à avaliação fisiológica invasiva no Projeto ANFIBIO (NCT05374694) foram classificados de acordo com a reserva de fluxo fracionada [(RFF)≤0,80] e o índice de resistência microcirculatória [(IRM) ≥25] na artéria descendente anterior esquerda em quatro grupos: índices normais, disfunção epicárdica isolada, disfunção microvascular isolada e disfunção combinada. Os índices de pressão, fluxo e resistência coronários foram comparados entre os grupos.

**Resultados:**

Um total de 130 pacientes foram finalmente incluídos. Uma diminuição gradual no fluxo coronário hiperêmico [(Q_cor_) em mL/min; índices normais: 387±192, epicárdico isolado: 278±153, microvascular isolado: 130±41, combinados: 96±33; p<0,001] e um aumento progressivo na resistência coronária total [(R_Total_) em unidades Wood (WU); índices normais: 238±139, epicárdico isolado: 373±167, microvascular isolado: 694±210, combinado: 999±342; p<0,001] foram observados à medida que mais compartimentos estavam envolvidos. Além disso, a disfunção epicárdica foi associada principalmente à redução da pressão coronária distal (Pd) e ao aumento da resistência epicárdica [(R_Epi_) em WU; índices normais: 25±18, epicárdico isolado: 145±113, microvascular isolado: 66±34, combinados: 389±282; p<0,001], enquanto a disfunção microvascular foi caracterizada por Pd preservado, mas Q_cor_ acentuadamente diminuído e resistência microvascular aumentada [(R_Micro_) em WU; índices normais: 213±124, epicárdico isolado: 228±73, microvascular isolado: 628±195, combinados: 610±137; p<0,001. A disfunção combinada compartilha mecanismos de disfunção epicárdica e microvascular.

**Conclusões:**

Os endotipos coronários exibem padrões hemodinâmicos distintos com mecanismos isquêmicos específicos relacionados à pressão e ao fluxo. A avaliação fisiológica integrada é essencial para a caracterização precisa do endotipo e para estratégias terapêuticas personalizadas.

## Graphical abstract

**Figura Central:**
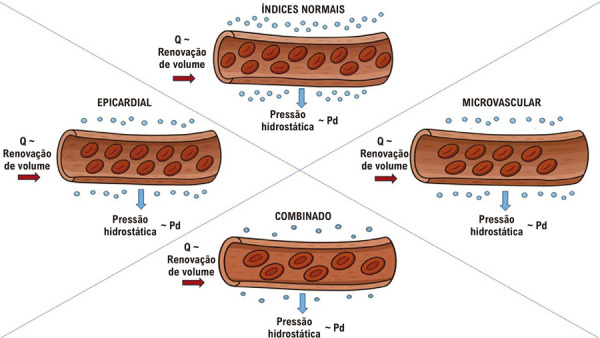
Caracterização Fisiológica de Endotipos Coronários: Possíveis Ligações com Isquemia Miocárdica

Fluxo coronário, pressão distal coronária e possíveis ligações com isquemia miocárdica. Q: fluxo coronário; Pd: pressão coronária distal. Q está relacionado à renovação do volume capilar e Pd à pressão hidrostática capilar. Na disfunção epicárdica, um Pd baixo se traduz em redução da pressão hidrostática capilar, enquanto a redução em Q é apenas ligeira. Na disfunção microvascular, Pd permanece preservado apesar de uma redução significativa em Q. A disfunção combinada mostra uma redução acentuada em Pd juntamente com Q gravemente comprometido.

## Introdução

Na prática clínica de rotina, um modelo bicompartimental é usado para estudar a regulação do fluxo sanguíneo coronário.^1 , 2^ O compartimento epicárdico, que compreende artérias ≥400µm, cuja função é distribuir o fluxo sanguíneo para os diferentes territórios, e o compartimento microvascular, que compreende artérias <400µm, arteríolas intermediárias e pequenas arteríolas, cuja função é regular a quantidade de fluxo sanguíneo que chega à rede capilar.^3 , 4^

A avaliação fisiológica de pacientes com suspeita de síndromes coronárias crônicas (SCC) depende cada vez mais de técnicas invasivas que permitem a avaliação funcional de cada compartimento coronário individualmente ou da circulação como um todo.^1 , 2^ O uso combinado de índices coronários, como “reserva de fluxo fracionada” (RFF), “reserva de fluxo coronário” (RFC) e “índice de resistência microcirculatória” (IRM), juntamente com o teste de vasoespasmo da artéria coronária (VAC), permite uma definição mais refinada dos endotipos coronários fisiológicos específicos do paciente.^5 - 8^

No entanto, a maioria dos estudos até o momento se concentrou na caracterização de endotipos associados à VAC ou disfunção microvascular em pacientes com angina e artérias coronárias não obstruídas (ANOCA).^8 , 9^ Em contraste, o perfil fisiológico de pacientes com isquemia relacionada à disfunção epicárdica, ou daqueles com disfunção epicárdica e microvascular combinadas, permanece amplamente inexplorado.^8 - 10^

Portanto, o presente estudo visa caracterizar sistematicamente todo o espectro de endotipos coronários fisiológicos, incluindo disfunção epicárdica isolada, disfunção microvascular isolada e disfunção combinada epicárdica e microvascular, em pacientes com suspeita de SCC, integrando índices de pressão, fluxo e resistência, e elucidar possíveis ligações mecanísticas com isquemia miocárdica nos diferentes padrões de disfunção coronária.

## Materiais e métodos

### População do estudo

Este é um subestudo do Projeto ANFIBIO, um estudo multicêntrico, descritivo e iniciado por investigadores, realizado em quatro centros espanhóis e concebido principalmente para identificar perfis de microRNA relacionados com padrões de envolvimento coronário. O desenho do Projeto ANFIBIO (NCT05374694) já foi descrito em outra publicação,^1 1^ e, em resumo, inclui pacientes com suspeita de angina encaminhados para angiografia coronária e eventual angioplastia, com os seguintes critérios de inclusão e exclusão.

#### Critérios de inclusão

1. Idade igual ou superior a 18 anos.
2. Pacientes com dor torácica sugestiva de angina são avaliados por um cardiologista e encaminhados para angiografia coronária e eventual angioplastia coronária.
3. Anormalidades ecocardiográficas que podem causar dor no peito, como doença valvar grave (por exemplo, estenose aórtica grave…), disfunção ventricular esquerda grave, hipertensão pulmonar grave, entre outras.
4. Consentimento informado.

#### Critérios de exclusão

1. Alergia ao contraste não suscetível à pré-medicação.
2. Asma brônquica grave ou intolerância à adenosina.
3. Bloqueio atrioventricular (≥2º grau) ou intolerância à acetilcolina.
4. Infarto agudo do miocárdio com elevação do segmento ST.
5. Infarto agudo do miocárdio sem elevação do segmento ST.
6. Choque cardiogênico.
7. Oclusão total de qualquer artéria coronária que impossibilite medições com fios-guia de pressão-temperatura.
8. Cirurgia prévia de revascularização do miocárdio.
9. Mulheres com possibilidade de gravidez.
10. Disfunção renal com taxa de filtração glomerular estimada <30 mL/min/1,73m^2^.
11. Incapacidade de compreender a natureza do estudo e/ou assinar o termo de consentimento livre e esclarecido.
12. Qualquer outra condição médica que, na opinião do pesquisador, possa acarretar problemas de segurança para os pacientes ou alterar os resultados do estudo.

O desenho do Projeto ANFIBIO classificou os pacientes de acordo com a presença de disfunção epicárdica e/ou microvascular, com base nos valores de RFF e IRM. A disfunção epicárdica foi definida como um valor de RFF ≤ 0,80 e a disfunção microvascular como um valor de IRM ≥ 25. A avaliação da artéria coronária descendente anterior esquerda (DAE) foi obrigatória em todos os pacientes, e outros vasos (principais ou secundários) foram avaliados apenas em caso de estenose coronária ≥ 30% ou isquemia induzida em testes de estresse, correspondentes aos seus territórios.^11^

**Figura 1:**
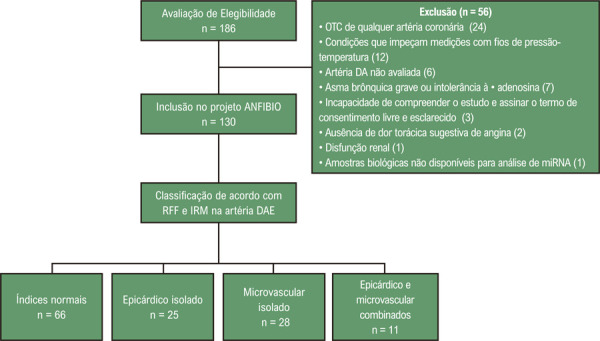
– Fluxograma do estudo. OTC: oclusão total crônica; DAE: artéria descendente anterior esquerda; RFF: reserva de fluxo fracionada; IRM: índice de resistência microcirculatória; miRNA: microRNA.

Portanto, para este subestudo, foi utilizada apenas a avaliação da DAE, disponível para todos os pacientes, reclassificando-os de acordo com os valores de RFF e IRM na DAE. Os diferentes grupos de estudo, de acordo com as possíveis combinações de valores de RFF e IRM, são apresentados na Tabela 1 . O fluxograma do estudo é apresentado na Figura 1 .

**Tabela 1:** – Grupos de estudo de acordo com a avaliação fisiológica invasiva

FFR e IRM	
Índices Normais (n=66)	RFF > 0,8 IRM < 25
Epicárdico isolado (n=25)	RFF ≤ 0,8 IRM < 25
Microvascular isolado (n=28)	RFF > 0,8 IRM ≥ 25
Combinação de Epicárdio e Microvascular (n=11)	RFF ≤ 0,8 IRM ≥ 25

RFF: reserva de fluxo fracionada; IRM: índice de resistência microvascular.

#### Procedimento de diagnóstico e medições

Resumidamente, o protocolo diagnóstico foi realizado da seguinte forma.^1 1^ Após a angiografia coronária diagnóstica, uma análise padronizada por angiografia coronária quantitativa (ACQ) utilizando o software CAAS 2000 foi realizada em cada centro. As medições fisiológicas invasivas foram realizadas de acordo com as recomendações.^1 , 2^ Um fio-guia com sensor de pressão e temperatura na ponta (Pressure Wire™ X guidewire 0.014’, Abbott, IL, EUA) foi utilizado. Após a administração de 200 µg de nitroglicerina, o sensor de pressão na ponta foi avançado até a porção médio-distal do vaso. A pressão aórtica em repouso (Pa) e a pressão coronária distal (Pd) foram obtidas, e os índices de repouso, Pd/Pa e “razão de ciclo completo em repouso” (RFR), foram determinados. Para medir a Tmn em repouso, bolus intracoronários de 3 mL de solução salina à temperatura ambiente foram injetados manualmente três vezes em sucessão (3 mL/s).

Em seguida, a hiperemia máxima foi induzida utilizando adenosina intravenosa (140 a 180 mg/kg/min) como vasodilatador, e três volumes adicionais de 3 mL de solução salina à temperatura ambiente foram administrados por via intracoronária para determinar o Tmn hiperêmico. Finalmente, o RFF, o IRM, o “IRM corrigido” (IRM_corr_), o RFC e a “razão de reserva resistiva” (RRR) foram determinados, utilizando o software Coroventis Coroflow (Coroventis AB, Uppsala, Suécia), em todos os centros.

Além disso, o “fluxo coronário” em repouso e hiperêmico (Q^cor^), e a “resistência coronária total” (R^Total^), a “resistência coronária epicárdica” (R_Epi_) e a “resistência coronária microvascular” (R_Micro_), sob hiperemia máxima, foram calculados por fórmulas descritas no Material Suplementar .^7 , 12 - 20^

Adicionalmente, a VAC foi avaliada em casos que apresentavam índices coronários normais ou disfunção microvascular isolada, através da administração intracoronária incremental de acetilcolina (2, 20 e 100 µg) na artéria coronária esquerda, seguindo um protocolo padronizado previamente descrito.^11^ O espasmo epicárdico foi definido como a presença de angina, alterações eletrocardiográficas e contração do diâmetro arterial ≥90%. O espasmo microvascular foi definido como a presença de angina, alterações eletrocardiográficas e contração do diâmetro arterial <90%.^1 , 2 , 11^

É importante destacar que um investigador dedicado foi responsável por registrar os valores de cada variável angiográfica e fisiológica, enquanto os operadores permaneceram alheios à atribuição desses valores.

## Análise estatística

As variáveis categóricas foram expressas em contagens (percentagens). A distribuição normal das variáveis contínuas foi verificada pelo teste de Kolmogorov-Smirnov. As variáveis contínuas foram expressas como média ± desvio padrão (DP) ou mediana com IIQ (intervalo interquartil), de acordo com sua distribuição. Independentemente da distribuição, Tmn e Q_cor_ foram expressos como média ± DP e mediana (IIQ). Para comparar variáveis categóricas, foram utilizados testes qui-quadrado ou testes exatos de Fisher, conforme apropriado. Para comparar variáveis contínuas entre os grupos de estudo, foi realizada uma análise de variância (ANOVA) de uma via. Quando efeitos significativos foram identificados, testes post-hoc com correção de Bonferroni foram aplicados para ajustar o nível de significância em comparações entre múltiplos grupos e reduzir o risco de erros do Tipo I. Além disso, para controlar possíveis variáveis de confusão na alocação aos grupos de estudo, foram empregados modelos lineares generalizados, incluindo sexo e presença de diabetes mellitus como covariáveis.

O nível de significância bicaudal foi definido como p < 0,05. Todas as análises foram realizadas utilizando o software SPSS Statistics 20.0 (SPSS Inc., Chicago, IL, EUA).

## Resultados

De um total de 186 pacientes avaliados para elegibilidade, cento e trinta pacientes foram recrutados para este subestudo do Projeto ANFIBIO. Após a reclassificação dos pacientes de acordo com os valores de RFF e IRM na DAE, a distribuição dos pacientes finalmente avaliados para esta subanálise foi a seguinte: a) índices normais (n=66); b) disfunção epicárdica isolada (n=25); c) disfunção microvascular isolada (n=28); e d) disfunção epicárdica e microvascular combinada (n=11) ( Figura 1 ).

### Características basais

As características basais estão descritas na Tabela 2 . A proporção de pacientes do sexo feminino foi maior no grupo com índices normais (p=0,002) em comparação com os outros grupos, e o hábito de fumar variou entre os grupos (p=0,022). Destaca-se a presença de uma prevalência diferente de diabetes entre os grupos, mostrando menor prevalência em pacientes com índices normais e disfunção microvascular, e maior prevalência em pacientes com disfunção epicárdica isolada e disfunção epicárdica combinada (p=0,021). Os modelos lineares generalizados não mostraram interações significativas em relação ao sexo (p=0,080) ou diabetes mellitus (p=0,106) na alocação aos grupos de estudo. Nenhuma outra diferença foi observada.

**Tabela 2:** – Características basais

	Índices Normais (n=66)	Epicárdico isolado (n=25)	Microvascular isolado (n=28)	Combinação de Epicárdio e Microvascular (n=11)	valor p
**Idade, (anos), média (DP)**	65,8 (10,7)	61,2 (13,9)	67,9 (8,2)	70,7 (7,0)	0,050
**Sexo feminino, n (%)**	33 (50,0%)	3 (12,0%)	6 (21,4%)	4 (36,4%)	0,002
**IMC, (Kg/m** ^**2**^ **), média (DP)**	28,5 (5,8)	28,7 (3,6)	27,2 (4,0)	28,9 (6,6)	0,650
**Hábito de fumar**					0,022
Não fumante, n (%)	32 (48,5%)	10 (40,0%)	9 (33,3%)	9 (81,8%)	
Ex-fumante, n (%)	24 (36,4%)	11 (44,0%)	17 (63,0%)	0 (0%)	
Fumante atual, n (%)	10 (15,2%)	4 (16,0%)	1 (3,7%)	2 (18,2%)	
**Alcoolismo ativo, n (%)**	2 (3,1%)	2 (8,0%)	0 (0%)	1 (9,1%)	0,373
**Hipertensão, n (%)**	44 (66,7%)	18 (72,0%)	22 (81,5%)	7 (63,6%)	0,514
**Dislipidemia, n (%)**	38 (57,6%)	17 (68,0%)	18 (66,7%)	7 (63,6%)	0,755
**Diabetes mellitus, n (%)**	13 (19,7%)	11 (44,0%)	6 (22,2%)	6 (54,5%)	0,021
**Histórico familiar de doença isquêmica do coração, n (%)**	5 (7,6%)	2 (8,0%)	2 (7,4%)	1 (9,1%)	0,998
**SCA anterior, n (%)**	15 (22,7%)	12 (48,0%)	7 (25,9%)	2 (18,2%)	0,068
**Insuficiência cardíaca congestiva, n (%)**	5 (7,6%)	0 (0%)	1 (3,7%)	2 (18,2%)	0,181
**Fibrilação atrial, n (%)**	10 (15,2%)	2 (8,0%)	2 (7,4%)	2 (18,2%)	0,601
**Doença cerebrovascular, n (%)**	4 (6,1%)	0 (0%)	1 (3,7%)	0 (0%)	0,513
**Anamnese por intervenção coronária percutânea, n (%)**	15 (22,7%)	10 (40,0%)	8 (29,6%)	2 (18,2%)	0,351
**Doença vascular periférica, n (%)**	1 (1,5%)	2 (8,0%)	3 (11,1%)	0 (0%)	0,166
**DPOC, n (%)**	1 (1,5%)	1 (4,0%)	1 (3,7%)	0 (0%)	0,804
**SAOS, n (%)**	4 (6,1%)	4 (16,0%)	3 (11,1%)	0 (0%)	0,313
**Histórico de sangramento grave, n (%)**	1 (1,5%)	0 (0%)	0 (0%)	0 (0%)	0,810
**TFG, (mL/min/1,73m** ^**2**^ **), média (DP)**	79,2 (15,1)	75,9 (16,8)	73,8 (17,3)	75,1 (12,5)	0,453
**Hematócrito, (%), média (DP)**	41,9 (3,5)	43,5 (4,3)	43,3 (4,3)	42,3 (4,8)	0,262
**FEVE, (%), média (DP)**	62,4 (7,7)	62,9 (7,0)	64,8 (6,3)	60,8 (11,4)	0,475

DP: desvio padrão; IMC: índice de massa corporal; DAC: doença cardíaca isquêmica; SCA: síndrome coronariana aguda; ICP: intervenção coronária percutânea; DPOC: doença pulmonar obstrutiva crônica; SAOS: apneia obstrutiva do sono; TFG: taxa de filtração glomerular; FEVE: fração de ejeção do ventrículo esquerdo.

### Características angiográficas e medidas fisiológicas

As características angiográficas e as medidas fisiológicas são descritas na Tabela 3 . A distribuição da localização das estenoses coronárias foi diferente entre os grupos, destacando-se uma prevalência superior de ausência de estenoses coronárias em pacientes com índices normais e disfunção microvascular isolada, em comparação com pacientes com disfunção epicárdica isolada e disfunção combinada (p<0,001). Em relação às medidas fisiológicas, sob hiperemia máxima, a Pd foi menor nos casos de disfunção epicárdica isolada e disfunção combinada (índices normais: 68 ± 16 mmHg vs. epicárdica isolada: 55 ± 15 mmHg vs. microvascular isolada: 75 ± 14 mmHg vs. combinada: 56 ± 15 mmHg; p<0,001) e a Tmn foi maior nos casos de disfunção microvascular isolada e disfunção combinada (índices normais: 0,20 ± 0,15 s vs. epicárdica isolada: 0,27 ± 0,12 s vs. microvascular isolada: 0,52 ± 0,20 s vs. combinada: 0,71 ± 0,32 s; p<0,001). Não houve diferenças em relação ao espasmo microvascular ou epicárdico.

**Tabela 3:** – Características angiográficas e medidas fisiológicas

	Índices Normais (n=66)	Epicárdico isolado (n=25)	Microvascular isolado (n=28)	Combinação de epicárdio e microvascular (n=11)	valor p
**Características angiográficas**					
**Dominância coronária**					0,897
Direita, n (%)	50 (75,8%)	21 (84,0%)	23 (82,1%)	9 (81,8%)	
Esquerda, n (%)	9 (13,6%)	3 (12,0%)	4 (14,3%)	1 (9,1%)	
Codominância, n (%)	7 (10,6%)	1 (4,0%)	1 (3,6%)	1 (9,1%)	
**Localização da estenose principal**					<0,001
- Artéria coronária principal esquerda, n (%)	0 (0%)	1 (4,0%)	0 (0%)	0 (0%)	
DAE proximal, n (%)	5 (7,6%)	7 (28,0%)	2 (7,1%)	4 (36,4%)	
DAE Meia, n (%)	16 (24,2%)	15 (60,0%)	10 (35,7%)	6 (54,5%)	
DAE distal, n (%)	1 (1,5%)	2 (8,0%)	3 (10,7%)	1 (9,1%)	
Ausência de estenose, n (%)	44 (66,7%)	0 (0%)	13 (46,4%)	0 (0%)	
**Percentagem de estenose, (QCA %) média (DP)**	28 (13)†, ‡, §	55 (14) *, ‡	42 (13) *, †, §	65 (15) *, ‡	<0,001
**Comprimento da lesão, (mm), média (DP)**	9 (4) †, §	18 (10) *	12 (8)	19 (5) *	0,001
**Diâmetro do vaso, (mm), média (DP)**	2.6 (1.0)	2,9 (0,6)	2,6 (0,6)	2,9 (0,7)	0,339
**Medidas fisiológicas**					
**Pa em repouso, (mm Hg), média (DP)**	87 (18)	93 (15)	89 (18)	89 (7)	0,608
**Pd em repouso, (mm Hg), média (DP)**	83 (18)	73 (16)	83 (18)	69 (17)	0,017
**Tmn em repouso, (s), média (DP) mediana (IIQ)**	0,60 (0,28) ‡, § 0,56 (0,41-0,78)	0,64 (0,30) ‡, § 0,53 (0,48-0,87)	1,05 (0,37) *, † 1,06 (0,70-1,40)	1,06 (0,33) *, † 1,12 (0,72-1,23)	<0,001
**Pa hiperêmico, (mm Hg), média (DP)**	75 (17)	84 (15)	83 (15)	86 (10)	0,017
**Pd hiperêmico, (mm Hg), média (DP)**	68 (16) †	55 (15) *, ‡	75 (14) †, §	56 (15) ‡	<0,001
**Hiperemia Tmn, (s), média (DP) mediana (IIQ)**	0,20 (0,15) ‡, § 0,17 (0,13-0,23)	0,27 (0,12) ‡, § 0,28 (0,18-0,36)	0,52 (0,20) *, †, § 0,49 (0,36-0,59)	0,71 (0,32) *, †, ‡ 0,69 (0,49-0,84)	<0,001
**Vasoespasmo da artéria coronária**					0,644
Espasmo microvascular, n (%)	4 (6,1%)	-	2 (7,1%)	-	
Espasmo epicárdico, n (%)	21 (31,8%)	-	7 (25,0%)	-	

DAE: artéria coronária descendente anterior esquerda; DP: desvio padrão; Pa: pressão aórtica; Pd: pressão coronária distal; Tmn: tempo médio de trânsito; IIQ: intervalo interquartil. ^∗^p<0:05 em comparação com o grupo “Índices Normais”. † p<0:05 em comparação com o grupo “Epicárdico Isolado”. ‡ p<0:05 em comparação com o grupo “Microvascular Isolado”. § p<0:05 em comparação com o grupo “Epicárdico e Microvascular Combinados”.

### Índices coronários

**Figura 2:**
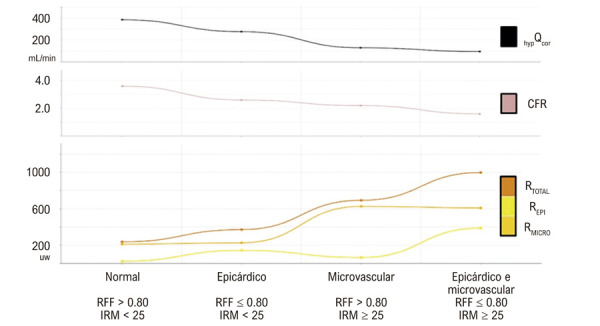
– Relações entre os valores médios do fluxo coronário, da reserva de fluxo coronário e das resistências coronárias. hipQcor: fluxo coronário hiperêmico; RFC: reserva de fluxo coronário; RTotal: resistência coronária total; REpi: resistência coronária epicárdica; RMicro: resistência coronária microvascular; RFF: reserva de fluxo fracionada; IRM: índice de resistência microcirculatória.

Na Tabela 4 , são apresentados os índices coronários. Em relação aos índices coronários, observaram-se diferenças não apenas no RFF e no IRM, utilizados para constituir os grupos, mas também em todos os demais índices coronários. Na Figura 2 , observa-se uma relação inversa entre o valor médio do Q_cor_ hiperêmico e do RFC com o valor médio do R^Total^. Além disso, observa-se que o R_Epi_ e o R_Micro_ aumentam nos casos de disfunção epicárdica e microvascular, respectivamente.

**Tabela 4:** – Índices coronários

	Índices Normais (n=66)	Epicárdico isolado (n=25)	Microvascular isolado (n=28)	Combinação de Epicárdio e Microvascular (n=11)	valor p
**RFF, média (DP)**	0,90 (0,04) †, §	0,66 (0,13) *, ‡	0,90 (0,05) †, §	0,65 (0,14) *, ‡	<0,001
**IRM, média (DP)**	12 (5) ‡, §	14 (4) ‡, §	36 (10) *, †	37 (8) *, †	<0,001
**RFC, média (DP)**	3.6 (1.4) †, ‡, §	2,6 (1,3) *	2,2 (0,8) *	1,6 (0,5) *	<0,001
**Pd/Pa, média (DP)**	0,94 (0,04) †, §	0,79 (0,12) *, ‡	0,93 (0,03) †, §	0,80 (0,13) *, ‡	<0,001
**RFR, média (DP)**	0,93 (0,02) †, §	0,76 (0,17) *, ‡	0,92 (0,04) †, §	0,70 (0,19) *, ‡	<0,001
**IRMcorr, média (DP)**	12 (5) ‡, §	11 (4) ‡, §	36 (11) *, †	31 (7) *, †	<0,001
**IRMcorr ≥23, n (%)**	2 (3,0%)	0 (0%)	28 (100%)	11 (100%)	<0,001
**RRR, média (DP)**	4.5 (2.0) ‡, §	4.0 (2.2) ‡, §	2,3 (0,9) *, †	2,2 (0,8) *, †	<0,001
**R**_**Total**_ **,(WU), média (DP) mediana (IIQ)**	238 (139) †, ‡, § 217 (152-269)	373 (167) *, ‡, § 360 (237-484)	694 (210) *, †, § 621 (530-773)	999 (342) *, †, ‡ 936 (800-1173)	<0,001
**R**_**Epi**_ **,(WU), média (DP) mediana (IIQ)**	25 (18) †, § 22 (11-33)	145 (113) *, ‡, § 111 (56-200)	66 (34)†, § 58 (40-97)	389 (282) *, †, ‡ 270 (204-532)	<0,001
**R**_**Micro**_ **,(WU), média (DP) mediana (IIQ)**	213 (124) ‡, § 193 (140-241)	228 (73) ‡, § 210 (177-276)	628 (195) *, † 571 (486-670)	610 (137) *, † 590 (466-732)	<0,001
**Q**_**cor**_ **em repouso, (mL/min), média (DP) mediana (IIQ)**	126 (71) ‡, § 108 (77-148)	117 (59) ‡ 113 (69-126)	66 (27) *, † 57 (43-86)	62 (21) * 54 (49-83)	<0,001
**Q**_**cor**_ **hiperêmico, (mL/min), média (DP) mediana (IIQ)**	387 (192) †, ‡, § 353 (261-471)	278 (153) *, ‡, § 214 (169-333)	130 (41) *, † 124 (102-166)	96 (33) *, † 87 (71-122)	<0,001


RFF: reserva de fluxo fracionada; DP: desvio padrão; IRM: índice de resistência microcirculatória; RFC: reserva de fluxo coronário; Pd: pressão coronária distal; Pa: pressão aórtica; RFR: razão de ciclo completo em repouso; IRMcorr: índice corrigido de resistência microcirculatória; RRR: razão de reserva resistiva; RTotal: resistência coronária total; WU: unidades Wood; IIQ: intervalo interquartil; REpi: resistência coronária epicárdica; RMicro: resistência coronária microvascular; Qcor: fluxo coronário. *p<0:05 em comparação com o grupo “Índices Normais”. † p<0:05 em comparação com o grupo “Epicárdico Isolado”. ‡ p<0:05 em comparação com o grupo “Microvascular Isolado”. § p<0:05 em comparação com o grupo “Epicárdico e Microvascular Combinados”.

## Discussão

Este subestudo fisiológico sugere a existência de padrões hemodinâmicos coronários distintos, ou endotipos coronários, com base na localização anatômica da disfunção coronária. Os principais achados podem ser resumidos da seguinte forma: a) houve um aumento progressivo no R_Total_ e uma diminuição correspondente no Q_cor_ hiperêmico e no RFC, à medida que o número de compartimentos afetados aumentava; b) a contribuição relativa do R_Epi_ e do R_Micro_ para o R_Total_ variou de acordo com o endotipo específico; e c) a disfunção epicárdica foi caracterizada principalmente pelo aumento do R_Epi_ e pela redução do Pd, enquanto a disfunção microvascular foi definida pelo aumento do R_Micro_ e pela redução do Q_cor_.

### Caracterização fisiológica em diferentes endotipos coronários

Nossos resultados demonstram uma redução gradual no Q_cor_ hiperêmico e na RFC de pacientes com fisiologia normal para aqueles com disfunção combinada, sugerindo um comprometimento progressivo da perfusão coronária à medida que mais compartimentos são afetados. Esse fenômeno, consistente com relatos anteriores sobre disfunção coronária multinível, tem sido associado a piores desfechos clínicos.^10 , 21 - 23^

Em paralelo, o R_Total_ apresentou um aumento progressivo entre os endotipos, corroborando ainda mais o conceito de restrição cumulativa do fluxo. Curiosamente, esse aumento no R_Total_ não teve origem uniforme, pois as respectivas contribuições do R_Epi_ e do R_Micro_ variaram dependendo do local da disfunção. Em pacientes com disfunção epicárdica isolada, o aumento no R_Total_ foi impulsionado predominantemente pela elevação do R_Epi_. Por outro lado, naqueles com disfunção microvascular isolada, o R_Total_ estava elevado devido a um aumento acentuado no R_Micro_, consistente com informações anteriores que descrevem o aumento do R_Micro_ como uma característica da disfunção microvascular coronária.^1 , 2 , 7^ Notavelmente, os pacientes no grupo de endotipos combinados apresentaram elevações em ambos os componentes de resistência (R_Epi_ e R_Micro_).

Esses achados ressaltam a importância de uma análise compartimental abrangente. Embora medidas globais, como a RFC ou o R_Total_, forneçam uma impressão geral da fisiologia coronária, elas podem obscurecer a etiologia subjacente se índices específicos de cada compartimento, como RFF, IRM, R_Epi_ ou R_Micro_, não forem considerados.^1 , 5 - 7^ Isso é relevante não apenas em pacientes com angina e achados angiográficos inconclusivos,^24^ que podem apresentar disfunção microvascular, mas também naqueles com disfunção epicárdica, nos quais a falha em identificar estenoses hemodinamicamente significativas pode resultar na retenção de uma revascularização potencialmente benéfica. Portanto, a identificação correta do local da disfunção permite que os médicos adaptem a terapia de forma mais eficaz, abordando a fisiopatologia subjacente específica, seja visando o tônus microvascular com vasodilatadores, revascularizando e otimizando a terapia medicamentosa para estenoses epicárdicas ou combinando ambas as abordagens em casos de disfunção mista.^1 , 2^

É importante ressaltar que a prevalência de VAC epicárdica e microvascular não diferiu significativamente entre os grupos, sugerindo que a isquemia miocárdica foi predominantemente impulsionada por anormalidades estruturais ou funcionais fixas, e não por reatividade dinâmica transitória,^8 , 25^ em nossa população.

#### Papel da pressão distal e do fluxo nos endotipos coronários: possíveis ligações com a isquemia

A pressão de perfusão (Pd) fornece informações essenciais sobre a Pd que impulsiona o sangue através da microcirculação e é diretamente influenciada pela gravidade das estenoses epicárdicas a montante.^6 , 13 , 14^Em nosso estudo, pacientes com disfunção epicárdica apresentaram uma redução significativa na Pd, indicando uma queda substancial de pressão no segmento estenótico, consistente com seus valores mais baixos de RFF. Essa redução na Pd leva à diminuição da pressão hidrostática capilar, o que pode comprometer a troca transcapilar de oxigênio e nutrientes do lúmen para o interstício e, em última instância, para os cardiomiócitos. Tal comprometimento da oferta de oxigênio ao miocárdio dependente da pressão pode desempenhar um papel central na gênese da isquemia nesse endotipo.^26^Notavelmente, isso ocorre apesar de apenas uma modesta redução no Q_cor_ hiperêmico, sugerindo que a Pd, em vez do volume de fluxo, é o principal fator limitante para o suprimento metabólico neste contexto.

Em contraste, a disfunção microvascular é caracterizada por valores de Pd preservados, mas uma redução acentuada no Q_cor_ hiperêmico. Essa redução reflete uma renovação prejudicada do volume sanguíneo capilar, limitando a renovação do fornecimento de oxigênio e substratos, apesar da Pd adequada.^27^Assim, um mecanismo dependente do fluxo emerge como o principal contribuinte para a isquemia neste contexto, onde a troca volumétrica é insuficiente para atender às demandas metabólicas do miocárdio.

Em pacientes com disfunção epicárdica e microvascular combinadas, tanto a Pd quanto o Q_cor_ estão gravemente reduzidos, o que implica a coexistência de limitações relacionadas à pressão e ao fluxo. Esse comprometimento duplo provavelmente resulta em uma carga isquêmica agravada, impulsionada por ambos os mecanismos.

Em geral, esses achados reforçam a necessidade de uma avaliação fisiológica integrada que avalie simultaneamente os parâmetros de pressão e fluxo coronários ( Ilustração Central ). Essa abordagem permite a distinção de mecanismos isquêmicos específicos de cada endotipo, dependentes da pressão na disfunção epicárdica e dependentes do fluxo na disfunção microvascular.^1 , 2^

## Limitações

Primeiro, esta é uma análise post-hoc de um estudo descritivo,^1 1^ que não foi especificamente concebido para o objetivo atual. Segundo, o pequeno tamanho da amostra de alguns grupos, particularmente o grupo com endotipo combinado, pode limitar a robustez de nossas conclusões. Portanto, os achados devem ser interpretados como geradores de hipóteses e necessitam de confirmação em estudos maiores. Terceiro, a classificação dos pacientes em diferentes grupos foi realizada de acordo com os critérios do desenho inicial do estudo, com base nos valores de RFF e IRM.^1 1^ No entanto, existem preocupações quanto à utilidade do uso do IRM para determinar o comprometimento da microcirculação em casos de disfunção epicárdica.^15^ Contudo, em nosso estudo, a classificação dos pacientes não foi afetada pelo uso do IRM em pacientes com valores de RFF ≤0,80, visto que nenhum dos pacientes teria sido reclassificado utilizando o IRM_corr_^19^ em caso de doença funcional epicárdica. Quarto, os resultados se aplicam apenas à DAE, sendo necessários estudos adicionais avaliando as artérias circunflexa esquerda e coronária direita.

## Conclusões

Os endotipos coronários definidos por disfunção epicárdica e/ou microvascular exibem diversos padrões hemodinâmicos. À medida que mais compartimentos são afetados, observa-se um aumento progressivo no RTotal com uma redução correspondente no Q_cor_ e no RFC. A disfunção epicárdica é caracterizada principalmente por uma redução significativa no Pd, enquanto a disfunção microvascular está associada principalmente a uma diminuição acentuada no Q_cor_. Esses achados destacam a importância de uma caracterização fisiológica integrada para identificar com precisão o local predominante da disfunção e orientar estratégias terapêuticas personalizadas.

## *Material suplementar

Material suplementar
