# Supplementary material for: Caracterização Fisiológica de Endotipos Coronários: Possíveis Ligações com Isquemia Miocárdica
Source: Arq Bras Cardiol. 2026 Jan 9;122(12):e20250340. [Article in Portuguese] doi: 10.36660/abc.20250340 (PMC12981356; doi:10.36660/abc.20250340)
Supplement: Material suplementar [file 0066-782x-abc-122-12-e20250340-suppl01.pdf]

**Supplementary material.**

We want to highlight that most of the following coronary indices are develop under the assumption that pressure in venous sinus (Pv) is ≈ 0 mmHg. Herein, we present their calculations using direct coronary measures. For some indices, we also expressed as a function of other coronary indices, in order to improve the understanding of these indices.

**- Coronary Flow ($Q_{cor}$)**[12]

$Q_{cor}$ was calculated using Tmn as follows:

$$Q_{cor} = 1 / Tmn$$

In addtition, $Q_{cor}$ can be calculated at rest or at maximal hyperemia to determinate resting or hyperemic $Q_{cor}$

$$_{rest}Q_{cor} = 1 / {}_{rest}Tmn \quad _{hyp}Q_{cor} = 1 / {}_{hyp}Tmn$$

Depending on the units to express the flow, the second term of the prior equation has to be multiplied by 1 mL or by $10^{-3}$ L to obtain mL/s or L/s or multiplied by 1 mL × [60 s / 1 min] or by $10^{-3}$ L × [60 s / 1 min] to obtain mL/min or L/min:

$$Q_{cor}\,(mL/s) = [1 / Tmn\,(s)] \times 1\,mL$$

$$Q_{cor}\,(L/s) = [1 / Tmn\,(s)] \times 10^{-3}\,L$$

$$Q_{cor}\,(mL/min) = [1 / Tmn\,(s)] \times 1\,mL \times [60\,s / 1\,min]$$

$$Q_{cor}\,(L/min) = [1 / Tmn\,(s)] \times 10^{-3}\,L \times [60\,s / 1\,min]$$

**- Fractional Flow Reserve (FFR)**[13,14]

FFR was calculated at maximal hyperemia as follows:

$$FFR = {}_{hyp}Pd \ / \ {}_{hyp}Pa$$

**- Ratio Distal Coronary Pressure / Aortic Pressure (Pd/Pa)**[13,14]

Pd/Pa was calculated at rest as follows:

$$Pd/Pa = {}_{rest}Pd \ / \ {}_{rest}Pa$$

- **Index of Microcirculatory Resistance (IMR)**[15]

IMR was calculated at maximal hyperemia as follows:

$$IMR = {}_{hyp}Pd \times {}_{hyp}Tmn$$

**- Coronary Flow Reserve (CFR)**[16,17]

CFR was calculated using Tmn values at rest and under maximal hyperemia as follows:

$$CFR = {}_{rest}Tmn \ / \ {}_{hyp}Tmn$$

- **Resting Full-Cycle Ratio (RFR)**[18]

RFR was obtained from Coroventis Coroflow system. RFR was calculated at rest and defined as:

$$RFR = \text{Lowest value of } {}_{rest}Pd \ / \ {}_{rest}Pa \text{ during the whole cardiac cycle}$$

**- Corrected IMR ($IMR_{corr}$)**[19]

Corrected IMR was calculated at maximal hyperemia, using the Yong's formula, as follows:

$$IMRcorr = {}_{hyp}Pa \times {}_{hyp}Tmn \times [(1.35 \times {}_{hyp}Pd \ / \ {}_{hyp}Pa) - 0.32$$

**- Resistive Reserve Ratio (RRR)**[20]

RRR was calculated using CRF values as well as Pd at rest and under maximal hyperemia as follows:

$$RRR = ({}_{rest}Tmn \ / \ {}_{hyp}Tmn) \times ({}_{rest}Pd \ / \ {}_{hyp}Pd) = CFR \times ({}_{rest}Pd \ / \ {}_{hyp}Pd)$$

**- Total Coronary Resistance ($R_{Total}$)**[7]

$R_{Total}$ was calculated at maximal hyperemia and expresssed in Wood units or mmHg/L/min as follows:

$$R_{Total} \ (WU) = {}_{hyp}Pa \ (mmHg) \ / \ {}_{hyp}Q_{cor} \ (L/min)$$

**- Epicardial Coronary Resistance ($R_{Epi}$)**[7]

$R_{Epi}$ was calculated at maximal hyperemia and expresssed in Wood units or mmHg/L/min as follows:

$$R_{Epi} \ (WU) = ({}_{hyp}Pa - {}_{hyp}Pd) \ (mmHg) \ / \ {}_{hyp}Q_{cor} \ (L/min)$$

**- Microvascular Coronary Resistance ($R_{Micro}$)**[7]

$R_{Micro}$ was calculated at maximal hyperemia and expresssed in Wood units or mmHg/L/min as follows:

$$R_{Micro} \ (WU) = {}_{hyp}Pd \ (mmHg) \ / \ {}_{hyp}Q_{cor} \ (L/min)$$
