# Supplementary material for: Caracterização Fisiológica de Endotipos Coronários: Possíveis Ligações com Isquemia Miocárdica
Source: Arq Bras Cardiol. 2026 Jan 9;122(12):e20250340. [Article in Portuguese] doi: 10.36660/abc.20250340 (PMC12981356; doi:10.36660/abc.20250340)
Supplement: Translation

# Translation: Physiological Characterization of Coronary Endotypes: Potential Links with Myocardial Ischemia

**DOI:** 10.36660/abc.20250340i

**Authors:** Lucía Matute-Blanco, Thalia Belmonte, Kristian Rivera, Marcos García-Guimaraes, Juan Casanova-Sandoval, Iván D. Benítez, Georgina Fuertes-Ferre, Ainhoa Pérez Guerrero, Raúl Millán Segovia, Fernando Worner, David De Gonzalo-Calvo, Diego Fernández-Rodríguez

**Affiliations:** 1 Espanha Arnau de Vilanova University Hospital, Lleida – Espanha; 2 Spain IRBLleida - Grup de Fisiologia i Patologia Cardiaca, Institut de Recerca Biomèdica de Lleida Fundació Dr. Pifarré, Lleida – Spain; 3 Spain IRBLleida - Translational Research in Respiratory Medicine Group, Institut de Recerca Biomèdica de Lleida Fundació Dr. Pifarré, Lleida – Spain; 4 Spain Centro de Investigación Biomédica en Red de Enfermedades Respiratorias (CIBERES), Instituto de Salud Carlos III, Madrid – Spain; 5 Spain Hospital Universitario de Cabueñes, Gijón, Asturias – Spain; 6 Spain Department of Basic Medical Sciences, Lleida Biomedical Research Institute (IRBLleida), University of Lleida, Lleida – Spain; 7 Spain Hospital Universitario Miguel Servet - Department of Cardiology, Zaragoza – Spain; 8 Spain Hospital Clínico Universitario Lozano Blesa - Department of Cardiology, Zaragoza – Spain; 9 Spain Hospital Universitario Son Espases - Department of Cardiology, Palma – Spain

**Keywords:** Coronary Circulation, Coronary Artery Disease, Myocardial Fractional Flow Reserve

## Abstract

**Background:**

Comprehensive physiological characterization of coronary endotypes remains limited, especially in epicardial dysfunction.

**Objectives:**

To describe hemodynamic profiles across the full spectrum of coronary endotypes and explore potential links with ischemia.

**Methods:**

Patients with suspected chronic coronary syndromes who underwent invasive physiological assessment in the ANFIBIO Project (NCT05374694) were classified according to fractional flow reserve [(FFR)≤0.80] and index of microcirculatory resistance [(IMR)≥25] in the left anterior descending artery into four groups: normal indices, isolated epicardial, isolated microvascular, and combined dysfunction. Coronary pressure, flow, and resistance indices were compared between groups.

**Results:**

A total of 130 patients were finally included. A gradual decrease in hyperemic coronary flow [(Q_cor_) in mL/min; normal indices: 387±192, isolated epicardial: 278±153, isolated microvascular: 130±41, combined: 96±33; p<0.001] and a progressive increase in total coronary resistance [(R_Total_) in Wood units (WU); normal indices: 238±139, isolated epicardial: 373±167, isolated microvascular: 694±210, combined: 999±342; p<0.001] were observed as more compartments were involved. Also, epicardial dysfunction was primarily associated with reduced distal coronary pressure (Pd) and elevated epicardial resistance [(R_Epi_) in WU; normal indices: 25±18, isolated epicardial: 145±113, isolated microvascular: 66±34, combined: 389±282; p<0.001], whereas microvascular dysfunction was characterized by preserved Pd but markedly decreased Q_cor_and increased microvascular resistance [(R_Micro_) in WU; normal indices: 213±124, isolated epicardial: 228±73, isolated microvascular: 628±195, combined: 610±137; p<0.001]. Combined dysfunction shares mechanisms of both epicardial and microvascular dysfunction.

**Conclusions:**

Coronary endotypes exhibit distinct hemodynamic patterns with specific pressure- and flow-related ischemic mechanisms. Integrated physiological assessment is essential for accurate endotype characterization and personalized therapeutic strategies.

## Introduction

In routine clinical practice, a bicompartmental model is used to study the regulation of coronary blood flow.^1 , 2^The epicardial compartment, which comprises arteries ≥400µm, whose function is to distribute blood flow to the different territories, and the microvascular compartment, which comprises arteries <400µm, intermediate arterioles, and small arterioles, whose function is to regulate the amount of blood flow reaching the capillary network.^3 , 4^

The physiological evaluation of patients with suspected chronic coronary syndromes (CCS) increasingly relies on invasive techniques that allow for the functional assessment of each coronary compartment individually or the circulation as a whole.^1 , 2^The combined use of coronary indices, such as “fractional flow reserve” (FFR), “coronary flow reserve” (CFR), and “index of microcirculatory resistance” (IMR), along with coronary artery vasospasm (CAV) testing, enables a more refined definition of patient-specific physiological coronary endotypes.^5 - 8^

However, most studies to date have focused on characterizing endotypes associated with CAV or microvascular dysfunction in patients with angina and non-obstructed coronary arteries (ANOCA).^8 , 9^In contrast, the physiological profiling of patients with ischemia related to epicardial dysfunction, or those with combined epicardial and microvascular dysfunction, remains largely underexplored.^8 - 10^

Therefore, the present study aims to systematically characterize the full spectrum of physiological coronary endotypes, including isolated epicardial, isolated microvascular, and combined epicardial and microvascular dysfunction, in patients with suspected CCS, by integrating pressure, flow, and resistance indices, and to elucidate potential mechanistic links with myocardial ischemia across the different patterns of coronary dysfunction.

## Materials and methods

### Study Population

This is a substudy of the ANFIBIO Project, a multicentric investigator-initiated and descriptive study conducted in four Spanish centers and primarily designed to identify miRNA profiles related to patterns of coronary involvement. The design of the ANFIBIO Project (NCT05374694) has been described elsewhere,^11^and in summary, it includes patients with suspected angina referred for coronary angiography and eventual angioplasty, with the following inclusion and exclusion criteria.

#### Inclusion Criteria

1. Age ≥ 18 years.

2. Patients with chest pain suggestive of angina evaluated by a cardiologist and referred for coronary angiography and eventual coronary angioplasty.

3. Echocardiographic abnormalities that could cause chest pain, such as severe valvular disease (e.g., severe aortic stenosis…), severe left ventricular dysfunction, severe pulmonary hypertension, among others.

4. Informed consent.

#### Exclusion Criteria

1. Contrast allergy not susceptible to premedication.

2. Severe bronchial asthma or adenosine intolerance.

3. Atrio-ventricular block (≥2nd degree) or acetylcholine intolerance.

4. Acute myocardial infarction with ST-segment elevation.

5. Acute myocardial infarction without ST-segment elevation.

6. Cardiogenic shock.

7. Total occlusion of any coronary artery that excludes measurements with pressure-temperature guidewires.

8. Previous coronary artery bypass grafting.

9. Women with the possibility of being pregnant.

10. Renal dysfunction with an estimated glomerular filtration rate <30 mL/min/1.73m^2^.

11. Inability to understand the nature of the study and/or sign informed consent.

12. Any other medical condition that, in the opinion of the researcher, may lead to safety issues for patients or may alter the results of the study.

The design of the ANFIBIO Project classified the patients according to the presence of epicardial and/or microvascular dysfunction, based on the FFR and the IMR values. Epicardial dysfunction was defined as whether the FFR value was ≤0.80, and microvascular dysfunction as whether the IMR value was ≥25. The evaluation of the left anterior descending (LAD) coronary artery was mandatory in all patients, and other vessels (main or secondary) were only evaluated in case of coronary stenosis ≥30% or inducible ischemia in stress tests, corresponding to their territories.^11^

Therefore, only the LAD evaluation, which was available in all patients, was used for the purpose of this substudy, reclassifying patients according to the FFR and IMR values in LAD. Different groups of study, according to the possible combinations of FFR and IMR values, are shown in Table 1 . The study flow chart is depicted in Figure 1 .

**Table 1:** – Study groups according to invasive physiological evaluation

FFR & IMR	
Normal Indices (n=66)	FFR > 0.8 IMR < 25
Isolated Epicardial (n=25)	FFR ≤ 0.8 IMR < 25
Isolated Microvascular (n=28)	FFR > 0.8 IMR ≥ 25
Combined Epicardial & Microvascular (n=11)	FFR ≤ 0.8 IMR ≥ 25

FFR: fractional flow reserve; IMR: index of microvascular resistance.

**Figure 1:** – Study flow chart. CTO: chronic total occlusion; LAD: left anterior descending; FFR: fractional flow reserve; IMR: index of microcirculatory resistance; miRNA: microRNA.

#### Diagnostic Procedure and Measurements

Briefly, the diagnostic protocol was carried out as follows.^11^After diagnostic coronary angiography, a standardized analysis by quantitative coronary angiography (QCA) powered by CAAS 2000 was performed at each center. Invasive physiological measurements were performed according to recommendations.^1 , 2^An intracoronary pressure and temperature sensor-tipped guidewire (Pressure Wire TM X guidewire 0.014’, Abbott, IL, USA) was used. After administration of 200µg of nitroglycerin, the tip pressure sensor was advanced to the mid-distal portion of the vessel. The resting aortic pressure (Pa) and distal coronary pressure (Pd) were obtained, and resting indices, Pd/Pa and “resting full-cycle ratio” (RFR) were determined. To measure the Tmn at rest, 3 mL of room-temperature saline intracoronary boluses were injected three times manually in succession (3 mL/s).

Then, maximal hyperemia was induced using adenosine iv (140 to 180 mg/kg/min) as a vasodilator, and three additional 3 mL of room-temperature saline intracoronary were administered to determine the hyperemic Tmn. Finally, the FFR, the IMR, the “corrected IMR” (IMR_corr_), the CFR, and the “resistive reserve ratio” (RRR) were determined, using the software Coroventis Coroflow (Coroventis AB, Uppsala, Sweden), in all centers.

Also, the resting and the hyperemic “coronary flow” (Q_cor_)_,_and the “total coronary resistance” (R_Total_), the “epicardial coronary resistance” (R_Epi_), and the “microvascular coronary resistance” (R_Micro_), under maximal hyperemia, were calculated by formulas depicted in the Supplementary Material .^7 , 12 - 20^

Additionally, CAV was assessed in cases presenting with normal coronary indices or isolated microvascular dysfunction, through incremental intracoronary administration of acetylcholine (2, 20, and 100µg) into the left coronary artery, following a standardized protocol previously described.^11^Epicardial spasm was defined as the presence of angina, electrocardiographic changes, and arterial diameter contraction ≥90%. Microvascular spasm was defined as the presence of angina, electrocardiographic changes, and arterial diameter contraction <90%.^1 , 2 , 11^

It is noteworthy that a dedicated investigator was responsible for recording the values of each angiographic and physiological variable, while the operators remained blinded to the assignment of these values.

## Statistical analysis

Categorical variables were expressed as counts (percentages). Continuous variables were explored for normal distribution using the Kolmogorov-Smirnov test. Continuous variables and expressed as mean ± standard deviation (SD) or median with interquartile range (IQR), according to their distribution. Irrespective of their distribution, the Tmn and Q_cor_were expressed as mean ± SD and median (IQR). To compare categorical variables, Chi-square tests or Fisher’s exact tests, as appropriate, were used. To compare continuous variables among the study groups, a one-way analysis of variance (ANOVA) was performed. When significant effects were identified, post-hoc tests with Bonferroni correction were applied to adjust the significance level in multiple group comparisons and reduce the risk of Type I errors. In addition, to control for potential confounding variables in the assignment to the study groups, generalized linear models were employed, including sex and the presence of diabetes mellitus as covariates.

A two-tailed significance level was set at p < 0.05. All analyses were performed using SPSS Statistics 20.0 software (SPSS Inc., Chicago, IL, USA).

## Results

From a total of 186 patients evaluated for eligibility, one hundred and thirty patients were recruited for this substudy of the ANFIBIO Project. After reclassifying patients according to FFR and IMR values in LAD, the distribution of patients finally evaluated for this subanalysis was the following: a) normal indices (n=66); b) isolated epicardial dysfunction (n=25); c) isolated microvascular dysfunction (n=28); and d) combined epicardial and microvascular dysfunction (n=11) ( Figure 1 ).

### Baseline characteristics

The baseline characteristics are described in Table 2 . The proportion of female patients was higher in the group with normal indices (p=0.002) as compared with other groups, and the smoking habit varied between the groups (p=0.022). Noteworthy is the presence of a different prevalence of diabetes between groups, showing less prevalence in patients with normal indices and microvascular dysfunction, and more prevalence in patients with isolated epicardial and combined epicardial dysfunction (p=0.021). Generalized linear models showed no significant interactions regarding sex (p=0.080) or diabetes mellitus (p=0.106) on the assignment to the study groups. No other differences were observed.

**Table 2:** – Baseline characteristics

	Normal Indices (n=66)	Isolated Epicardial (n=25)	Isolated Microvascular (n=28)	Combined Epicardial and Microvascular (n=11)	p value
**Age, (years), mean (SD)**	65.8 (10.7)	61.2 (13.9)	67.9 (8.2)	70.7 (7.0)	0.050
**Female sex, n (%)**	33 (50.0%)	3 (12.0%)	6 (21.4%)	4 (36.4%)	0.002
**BMI, (Kg/m** ^**2**^ **), mean (SD)**	28.5 (5.8)	28.7 (3.6)	27.2 (4.0)	28.9 (6.6)	0.650
**Smoking habit**					0.022
Not smoker, n (%)	32 (48.5%)	10 (40.0%)	9 (33.3%)	9 (81.8%)	
Prior smoker, n (%)	24 (36.4%)	11 (44.0%)	17 (63.0%)	0 (0%)	
Current smoker, n (%)	10 (15.2%)	4 (16.0%)	1 (3.7%)	2 (18.2%)	
**Active alcoholism, n (%)**	2 (3.1%)	2 (8.0%)	0 (0%)	1 (9.1%)	0.373
**Hypertension, n (%)**	44 (66.7%)	18 (72.0%)	22 (81.5%)	7 (63.6%)	0.514
**Dyslipidemia, n (%)**	38 (57.6%)	17 (68.0%)	18 (66.7%)	7 (63.6%)	0.755
**Diabetes mellitus, n (%)**	13 (19.7%)	11 (44.0%)	6 (22.2%)	6 (54.5%)	0.021
**Familial history of IHD, n (%)**	5 (7.6%)	2 (8.0%)	2 (7.4%)	1 (9.1%)	0.998
**Prior ACS, n (%)**	15 (22.7%)	12 (48.0%)	7 (25.9%)	2 (18.2%)	0.068
**Congestive heart failure, n (%)**	5 (7.6%)	0 (0%)	1 (3.7%)	2 (18.2%)	0.181
**Atrial fibrillation, n (%)**	10 (15.2%)	2 (8.0%)	2 (7.4%)	2 (18.2%)	0.601
**Cerebrovascular disease, n (%)**	4 (6.1%)	0 (0%)	1 (3.7%)	0 (0%)	0.513
**Prior PCI, n (%)**	15 (22.7%)	10 (40.0%)	8 (29.6%)	2 (18.2%)	0.351
**Peripheral vascular disease, n (%)**	1 (1.5%)	2 (8.0%)	3 (11.1%)	0 (0%)	0.166
**COPD, n (%)**	1 (1.5%)	1 (4.0%)	1 (3.7%)	0 (0%)	0.804
**OSA, n (%)**	4 (6.1%)	4 (16.0%)	3 (11.1%)	0 (0%)	0.313
**History of major bleeding, n (%)**	1 (1.5%)	0 (0%)	0 (0%)	0 (0%)	0.810
**GFR, (mL/min/1.73m** ^**2**^ **), mean (SD)**	79.2 (15.1)	75.9 (16.8)	73.8 (17.3)	75.1 (12.5)	0.453
**Hematocrit, (%), mean (SD)**	41.9 (3.5)	43.5 (4.3)	43.3 (4.3)	42.3 (4.8)	0.262
**LVEF, (%), mean (SD)**	62.4 (7.7)	62.9 (7.0)	64.8 (6.3)	60.8 (11.4)	0.475

SD: standard deviation; BMI: body mass index; IHD: ischemic heart disease; ACS: acute coronary syndrome; PCI: percutaneous coronary intervention; COPD: chronic obstructive pulmonary disease; OSA: obstructive sleep apnea; GFR: glomerular filtration rate; LVEF: left ventricular ejection fraction.

### Angiographic characteristics and physiological measurements

Angiographic characteristics and physiological measurements are described in Table 3 . The distribution of the location of coronary stenoses was different between groups, highlighting a superior prevalence of absence of coronary stenoses in patients with normal indices and isolated microvascular dysfunction, as compared with patients with isolated epicardial and combined dysfunction (p<0.001).

**Table 3:** – Angiographic characteristics and physiological measurements

	Normal Indices (n=66)	Isolated Epicardial (n=25)	Isolated Microvascular (n=28)	Combined Epicardial and Microvascular (n=11)	p value
**Angiographic characteristics**					
**Coronary dominance**					0.897
Right, n (%)	50 (75.8%)	21 (84.0%)	23 (82.1%)	9 (81.8%)	
Left, n (%)	9 (13.6%)	3 (12.0%)	4 (14.3%)	1 (9.1%)	
Codominance, n (%)	7 (10.6%)	1 (4.0%)	1 (3.6%)	1 (9.1%)	
**Location of main stenosis**					<0.001
Left main coronary artery, n (%)	0 (0%)	1 (4.0%)	0 (0%)	0 (0%)	
Proximal LAD, n (%)	5 (7.6%)	7 (28.0%)	2 (7.1%)	4 (36.4%)	
Mid LAD, n (%)	16 (24.2%)	15 (60.0%)	10 (35.7%)	6 (54.5%)	
Distal LAD, n (%)	1 (1.5%)	2 (8.0%)	3 (10.7%)	1 (9.1%)	
Absence of stenosis, n (%)	44 (66.7%)	0 (0%)	13 (46.4%)	0 (0%)	
**Percentage of stenosis, (QCA %), mean (SD)**	28 (13) †, ‡, §	55 (14) *, ‡	42 (13) *, †, §	65 (15) *, ‡	<0.001
**Length of the lesion, (mm), mean (SD)**	9 (4) †, §	18 (10) *	12 (8)	19 (5) *	0.001
**Vessel diameter, (mm), mean (SD)**	2.6 (1.0)	2.9 (0.6)	2.6 (0.6)	2.9 (0.7)	0.339
**Physiologic measurements**					
**Resting Pa, (mm Hg), mean (SD)**	87 (18)	93 (15)	89 (18)	89 (7)	0.608
**Resting Pd, (mm Hg), mean (SD)**	83 (18)	73 (16)	83 (18)	69 (17)	0.017
**Resting Tmn, (s), mean (SD) median (IQR)**	0.60 (0.28) ‡, § 0.56 (0.41-0.78)	0.64 (0.30) ‡, § 0.53 (0.48-0.87)	1.05 (0.37) *, † 1.06 (0.70-1.40)	1.06 (0.33) *, † 1.12 (0.72-1.23)	<0.001
**Hyperemic Pa, (mm Hg), mean (SD)**	75 (17)	84 (15)	83 (15)	86 (10)	0.017
**Hyperemic Pd, (mm Hg), mean (SD)**	68 (16) †	55 (15) *, ‡	75 (14) †, §	56 (15) ‡	<0.001
**Hyperemic Tmn, (s), mean (SD) median (IQR)**	0.20 (0.15) ‡, § 0.17 (0.13-0.23)	0.27 (0.12) ‡, § 0.28 (0.18-0.36)	0.52 (0.20) *, †, § 0.49 (0.36-0.59)	0.71 (0.32) *, †, ‡ 0.69 (0.49-0.84)	<0.001
**Coronary artery vasospasm**					0.644
Microvascular spasm, n (%)	4 (6.1%)	-	2 (7.1%)	-	
Epicardial spasm, n (%)	21 (31.8%)	-	7 (25.0%)	-	

SD: standard deviation; Pa: aortic pressure; Pd: distal coronary pressure; Tmn: mean transit time; IQR: interquartile range. *p<0.05 compared with the “Normal Indices” group. † p<0.05 compared with “Isolated Epicardial” group. ‡ p<0.05 compared with “Isolated Microvascular” group. § p<0.05 compared with “Combined Epicardial and Microvascular” group.

Regarding physiological measurements, under maximal hyperemia, Pd was lower in case of isolated epicardial dysfunction and combined dysfunction (normal indices: 68 ± 16 mm Hg vs. isolated epicardial: 55 ± 15 mm Hg vs. isolated microvascular: 75 ± 14 mm Hg vs. combined: 56 ± 15 mm Hg; p<0.001) and Tmn was higher in case of isolated microvascular dysfunction and combined dysfunction (normal indices: 0.20 ± 0.15 s vs. isolated epicardial: 0.27 ± 0.12 s vs. isolated microvascular: 0.52 ± 0.20 s vs. combined: 0.71 ± 0.32 s; p<0.001). There were no differences regarding microvascular or epicardial spasm.

### Coronary indices

In Table 4 , coronary indices are depicted. Regarding coronary indices, there were differences, not only in the FFR and IMR that were used to constitute the groups, but also in all the other coronary indices. In Figure 2 , we observe an inverse relationship between the mean value of the hyperemic Q_cor_and CFR with the mean value of R_Total_. Moreover, we observe that R_Epi_and R_Micro_increase in cases of epicardial and microvascular dysfunction, respectively.

**Table 4:** – Coronary indices

	Normal Indices (n=66)	Isolated Epicardial (n=25)	Isolated Microvascular (n=28)	Combined Epicardial and Microvascular (n=11)	p value
**FFR, mean (SD)**	0.90 (0.04) †, §	0.66 (0.13) *, ‡	0.90 (0.05) †, §	0.65 (0.14) *, ‡	<0.001
**IMR, mean (SD)**	12 (5) ‡, §	14 (4) ‡, §	36 (10) *, †	37 (8) *, †	<0.001
**CFR, mean (SD)**	3.6 (1.4) †, ‡, §	2.6 (1.3) *	2.2 (0.8) *	1.6 (0.5) *	<0.001
**Pd/Pa, mean (SD)**	0.94 (0.04) †, §	0.79 (0.12) *, ‡	0.93 (0.03) †, §	0.80 (0.13) *, ‡	<0.001
**RFR, mean (SD)**	0.93 (0.02) †, §	0.76 (0.17) *, ‡	0.92 (0.04) †, §	0.70 (0.19) *, ‡	<0.001
**IMRcorr, mean (SD)**	12 (5) ‡, §	11 (4) ‡, §	36 (11) *, †	31 (7) *, †	<0.001
**IMRcorr ≥23, n (%)**	2 (3.0%)	0 (0%)	28 (100%)	11 (100%)	<0.001
**RRR, mean (SD)**	4.5 (2.0) ‡, §	4.0 (2.2) ‡, §	2.3 (0.9) *, †	2.2 (0.8) *, †	<0.001
**R**_**Total**_ **, (WU), mean (SD) median (IQR)**	238 (139) †, ‡, § 217 (152-269)	373 (167) *, ‡, § 360 (237-484)	694 (210) *, †, § 621 (530-773)	999 (342) *, †, ‡ 936 (800-1173)	<0.001
**R**_**Epi**_ **, (WU), mean (SD) median (IQR)**	25 (18) †, § 22 (11-33)	145 (113) *, ‡, § 111 (56-200)	66 (34) †, § 58 (40-97)	389 (282) *, †, ‡ 270 (204-532)	<0.001
**R**_**Micro**_ **, (WU), mean (SD) median (IQR)**	213 (124) ‡, § 193 (140-241)	228 (73) ‡, § 210 (177-276)	628 (195) *, † 571 (486-670)	610 (137) *, † 590 (466-732)	<0.001
**Resting Q**_**cor**_ **, (mL/min), mean (SD) median (IQR)**	126 (71) ‡, § 108 (77-148)	117 (59) ‡ 113 (69-126)	66 (27) *, † 57 (43-86)	62 (21) * 54 (49-83)	<0.001
**Hyperemic Q**_**cor**_ **, (mL/min), mean (SD) median (IQR)**	387 (192) †, ‡, § 353 (261-471)	278 (153) *, ‡, § 214 (169-333)	130 (41) *, † 124 (102-166)	96 (33) *, † 87 (71-122)	<0.001

FFR: fractional flow reserve; SD: standard deviation; IMR: index of microcirculatory resistance; CFR: coronary flow reserve; Pd: distal coronary pressure; Pa: aortic pressure; RFR: resting full-cycle ratio; IMRcorr: corrected index of microcirculatory resistance; RRR: resistive reserve ratio; RTotal: total coronary resistance; WU: Wood units; IQR: interquartile range; REpi: epicardial coronary resistance; RMicro: microvascular coronary resistance; Qcor: coronary flow. * p<0.05 compared with the “Normal Indices” group. † p<0.05 compared with “Isolated Epicardial” group. ‡ p<0.05 compared with “Isolated Microvascular” group. § p<0.05 compared with “Combined Epicardial and Microvascular” group.

**Figure 2:** – Relationships between mean values of Coronary Flow, Coronary Flow Reserve, and Coronary Resistances. hypQcor: hyperemic coronary flow; CFR: coronary flow reserve; RTotal: total coronary resistance; REpi: epicardial coronary resistance; RMicro: microvascular coronary resistance; FFR: fractional flow reserve; IMR: index of microcirculatory resistance.

## Discussion

This physiological substudy suggests the existence of distinct hemodynamic coronary patterns or coronary endotypes, based on the anatomical location of coronary dysfunction. The main findings can be summarized as follows: a) there was a progressive increase in R_Total_and a corresponding decrease in hyperemic Q_cor_and CFR, as the number of affected compartments increased; b) the relative contribution of R_Epi_and R_Micro_to R_Total_varied according to the specific endotype; and c) epicardial dysfunction was primarily characterized by increased R_Epi_and reduced Pd, while microvascular dysfunction was defined by elevated R_Micro_and reduced Q_cor_.

### Physiological characterization across coronary endotypes

Our results demonstrate a stepwise reduction in hyperemic Q_cor_and CFR from patients with normal physiology to those with combined dysfunction, suggesting a progressive impairment in coronary perfusion as more compartments become involved. This phenomenon, consistent with previous reports on multilevel coronary dysfunction, has been associated with worse clinical outcomes.^10 , 21 - 23^

In parallel, R_Total_exhibited a progressive increase across endotypes, further substantiating the concept of cumulative flow restriction. Interestingly, this increase in R_Total_was not uniform in its origin because the respective contributions of R_Epi_and R_Micro_varied depending on the site of dysfunction. In patients with isolated epicardial dysfunction, the rise in R_Total_was driven predominantly by the elevation in R_Epi_. Conversely, in those with isolated microvascular dysfunction, R_Total_was elevated due to a marked increase in R_Micro_, consistent with previous information describing increased R_Micro_as a hallmark of coronary microvascular dysfunction.^1 , 2 , 7^Notably, patients in the combined endotype group exhibited elevations in both resistance components (R_Epi_and R_Micro_).

These findings underscore the importance of a comprehensive compartmental analysis. While global measurements, such as CFR or R_Total,_provide a general impression of coronary physiology, they may obscure the underlying etiology if compartment-specific indices, such as FFR, IMR, R_Epi,_or R_Micro,_are not considered.^1 , 5 - 7^This is relevant not only in patients with angina and inconclusive angiographic findings,^24^who may have microvascular dysfunction, but also in those with epicardial dysfunction, in whom failing to identify hemodynamically significant stenoses may result in withholding potentially beneficial revascularization. Therefore, the correct identification of the site of dysfunction enables clinicians to tailor therapy more effectively, addressing the specific underlying pathophysiology, whether by targeting microvascular tone with vasodilators, revascularizing and optimizing medical therapy for epicardial stenoses, or combining both approaches in cases of mixed dysfunction.^1 , 2^

Importantly, the prevalence of both epicardial and microvascular CAV did not differ significantly between groups, suggesting that myocardial ischemia was predominantly driven by fixed structural or functional abnormalities, rather than by transient dynamic reactivity,^8 , 25^in our population.

### Role of distal pressure and flow in coronary endotypes: potential links with ischemia

Pd provides essential insight into the perfusion pressure driving blood through the microcirculation and is directly influenced by the severity of upstream epicardial stenosis.^6 , 13 , 14^In our study, patients with epicardial dysfunction exhibited a significant reduction in Pd, indicating a substantial pressure drop across the stenotic segment, consistent with their lower FFR values. This reduction in Pd leads to decreased capillary hydrostatic pressure, which may compromise the transcapillary exchange of oxygen and nutrients from the lumen to the interstitium and ultimately to the cardiomyocytes. Such pressure-dependent impairment of myocardial oxygen delivery may play a central role in the genesis of ischemia in this endotype.^26^Notably, this occurs despite only a modest decline in hyperemic Q_cor_, suggesting that perfusion pressure, rather than flow volume, is the primary limiting factor for metabolic supply in this context.

In contrast, microvascular dysfunction is characterized by preserved Pd values but a marked reduction in hyperemic Q_cor_. This reduction reflects an impaired turnover of capillary blood volume, limiting the renewal of oxygen and substrate delivery despite adequate perfusion pressure.^27^Thus, a flow-dependent mechanism emerges as the dominant contributor to ischemia in this setting, where volumetric exchange is insufficient to meet myocardial metabolic demands.

In patients with combined epicardial and microvascular dysfunction, both Pd and Q_cor_are severely reduced, implicating the coexistence of pressure- and flow-related limitations. This dual compromise likely results in a compounded ischemic burden driven by both mechanisms.

Overall, these findings support the need for an integrated physiological assessment that concurrently evaluates coronary pressure and flow parameters ( Central Illustration ). This approach enables the distinction of endotype-specific ischemic mechanisms, pressure-dependent in epicardial dysfunction and flow-dependent in microvascular dysfunction.^1 , 2^

### Limitations

First, this is a post-hoc analysis of a descriptive study,^11^which was not specifically designed for the current aim. Second, the small sample size of some groups, particularly the combined endotype group, may limit the robustness of our conclusions. Therefore, the findings should be interpreted as hypothesis-generating and warrant confirmation in larger studies. Third, the classification of patients into different groups was carried out according to the criteria of the initial study design, according to the FFR and IMR values.^11^However, there are concerns about the usefulness of using the IMR to determine the affectation of the microcirculation in case of epicardial dysfunction.^15^However, in our study, the classification of patients was not affected by using the IMR in patients with FFR values ≤ 0.80, since none of the patients would have been reclassified using the IMR_corr_^19^in the case of epicardial functional disease. Fourth, results only apply to LAD, requiring further studies evaluating left circumflex and right coronary arteries.

## Conclusions

Coronary endotypes defined by epicardial and/or microvascular dysfunction exhibit diverse hemodynamic patterns. As more compartments became affected, a progressive increase in R_Total_with a corresponding reduction in Q_cor_and CFR is observed. Epicardial dysfunction is primarily characterized by a significant reduction in Pd, whereas microvascular dysfunction is mainly associated with a marked decrease in Q_cor_. These findings highlight the importance of an integrated physiological characterization to accurately identify the predominant site of dysfunction and to guide personalized therapeutic strategies.

## *Supplemental Materials

Supplementary material
